# A Novel Algorithm Based on the Pixel-Entropy for Automatic Detection of Number of Lanes, Lane Centers, and Lane Division Lines Formation

**DOI:** 10.3390/e20100725

**Published:** 2018-09-21

**Authors:** Fernando Hermosillo-Reynoso, Deni Torres-Roman, Jayro Santiago-Paz, Julio Ramirez-Pacheco

**Affiliations:** 1 CINVESTAV IPN Department of Electrical Engineering and Computer Sciences, Telecommunications Section, 45019 Zapopan, Jalisco, Mexico; 2 Department of Basic Sciences and Engineering (DCBel), University of Caribe, 77528 Cancún, Q. Roo, Mexico

**Keywords:** Shannon entropy, Tsallis entropy, entropic index q, automatic lane detection, surveillance systems, dtw, k-means, time series

## Abstract

Lane detection for traffic surveillance in intelligent transportation systems is a challenge for vision-based systems. In this paper, a novel pixel-entropy based algorithm for the automatic detection of the number of lanes and their centers, as well as the formation of their division lines is proposed. Using as input a video from a static camera, each pixel behavior in the gray color space is modeled by a time series; then, for a time period τ, its histogram followed by its entropy are calculated. Three different types of theoretical pixel-entropy behaviors can be distinguished: (1) the pixel-entropy at the lane center shows a high value; (2) the pixel-entropy at the lane division line shows a low value; and (3) a pixel not belonging to the road has an entropy value close to zero. From the road video, several small rectangle areas are captured, each with only a few full rows of pixels. For each pixel of these areas, the entropy is calculated, then for each area or row an entropy curve is produced, which, when smoothed, has as many local maxima as lanes and one more local minima than lane division lines. For the purpose of testing, several real traffic scenarios under different weather conditions with other moving objects were used. However, these background objects, which are out of road, were filtered out. Our algorithm, compared to others based on trajectories of vehicles, shows the following advantages: (1) the lowest computational time for lane detection (only 32 s with a traffic flow of one vehicle/s per-lane); and (2) better results under high traffic flow with congestion and vehicle occlusion. Instead of detecting road markings, it forms lane-dividing lines. Here, the entropies of Shannon and Tsallis were used, but the entropy of Tsallis for a selected *q* of a finite set achieved the best results.

## 1. Introduction

The global trend toward urbanization has experienced a worrying growth over the last 30 years, contributing to make cities socially, economically and environmentally unsustainable. One major serious aspect of urbanization is transportation, which includes traffic congestion, longer commutes, inadequate public transport, high infrastructure maintenance costs, environmental impacts and poor safety. Consequently, there has been an increasing requirement of developing intelligent systems with the purpose of improving the quality, rentability and use of the transportation in urban areas [1].

For smart cities applications, Intelligent Transportation Systems (ITS) have been developed. Applications such as traffic control systems, Closed-Circuit Television (CCTV) security systems, speed cameras, plate recognition technologies, and automatic lane detection are some examples of ITS. The surveillance of vehicular traffic using road-side static cameras and computer vision are important tools that allows us to observe traffic in real time, and identify, count and classify vehicles [2].

An important key part of the intelligent transportation systems (ITS) framework is VANET (Vehicular ad-hoc network) [3], which is composed of vehicles and infrastructure communicated mainly by Vehicle-to-Infrastructure (V2I) and Vehicle-to-Vehicle (V2V) [4]. They use wireless technologies, and allow the transmission/reception of alerts and warnings for hazardous situations (e.g., traffic jam, accidents, change of dynamic lanes, etc.), related to the local traffic.

The purpose of this paper is to address a particular case study of ITS: automatic lane detection through an analysis of pixel-entropy and Dynamic Time Warping (DTW) [5] using a traffic surveillance camera. Lane detection plays an important role in the modeling of highways, leading to the generation of traffic statistical data (e.g., vehicle load, speed, volume, etc.), as well as driving behavior and traffic collision detection.

First, an estimation of the entropic index *q* for the Tsallis entropy is performed as the procedure in [6] explains, followed by the automatic selection of *M* pixel rows equally spaced based on [7]. Second, each pixel of the *M* rows is modeled by a time series and then the histogram of each of these pixels and their entropy are calculated. Therefore, for each of these *M* rows, we obtain an entropy vector. Third, for each entropy vector, a peak finder algorithm is performed. Fourth, the entropy vectors corresponding to the selected *M* rows contain peaks representing the highest values or lane centers; these peaks are matched to each other and tracked according to the DTW algorithm to extract the lane centers and lane division lines. Finally, lanes are fitted by a second-order polynomial to reduce the complexity of the lanes.

The remainder of this paper is organized as follows: A review of the related work on automatic lane detection is presented in Section 2. Section 3 introduces the mathematical background, including entropy and DTW algorithm. In Section 4, the proposed method for lane detection based on the pixel-entropy is presented. A qualitative and quantitative analysis of the algorithm performance is in Section 5. The results are discussed in Section 6. Finally, Section 7 outlines the conclusions and future work.

## 2. Related Work

The easiest way to set up lane positions is geometrically, during system installation; nevertheless, more recent developments in video surveillance points towards the use of visual sensors such as Pan–Tilt–Zoom (PTZ) cameras [8,9], which are able to change their configuration to enhance the monitoring capabilities in such a way that, for the lane detection, the number of lanes and their position are dynamically changed.

Researchers have addressed the problem of lanes detection into two main classes: sensor-based methods (e.g., radar, laser sensors, etc.) and vision-based methods (e.g., road markings detection and trajectories clustering). Major advantages of sensor-based methods include higher scanning distances of up to 100 m, and robustness against weather conditions. However, inside of tunnels, the detection performance decreases, and its lane position estimation accuracy is usually lower than vision-based methods. Therefore, most of the recent research has focused on developing vision-based systems, and consequently we only focus on these methods.

*Lane marking-based methods*. Kim [10] used random sample consensus in conjunction with a particle filter for the lane marking extraction, and finally a probabilistic clustering algorithm to perform the lane detection. Daigavane and Bajaj [11] proposed an approach based on the edge detection, using ant colony optimization to link the edges that were disunited, and then applied the Hough transform for lane extraction. Lane marking-based methods provide a high performance solution of the lane detection problem mainly for Advanced Driver-Assistance Systems (ADAS) [12]. However, one of the major disadvantages is that lane markings are not always clearly visible due to the print wear off and to the image clarity by changes in environmental conditions.

*Trajectory clustering-based methods*. Melo et al. [13] proposed a method through a vehicle trajectory analysis with occlusion handling. First, vehicles are detected each frame by an adaptive smoothness algorithm for building a background model; then, Kalman filter is used to track vehicles trajectories which are represented by a low degree polynomial; and, finally, the lane centers are extracted by k-means clusters of trajectories. Ren et al. [14] proposed an enhanced trajectory-based framework for lane centers detection with a fast extraction of trajectories via vehicle feature points which are tracked by the pyramidal Kanade–Lucas–Tomasi algorithm. Then, trajectories are clustered by a modified k-means algorithm with Hausdorff distance for a fast trajectory extraction achieving a high accurate system. Trajectory clustering-based methods perform better than lane marking-based methods overcoming certain weaknesses. The major disadvantage of trajectory-based methods is that they require a set with a high number of well-formed trajectories on each lane which cannot be reached in short periods of time.

Motivated by the disadvantages and limitations of the traditional lane marking-based and trajectory-based methods mentioned in this section, we propose an algorithm for lane center detection and lane division lines formation.

By analyzing previous work, we conclude that, although lane markings-based methods and trajectory-based methods perform well in the lane detection, several issues related to their implementation are still unsolved:

1. Robustness against environmental conditions changes;
2. Low computational and convergence time;
3. Accurately extraction of both lane division lines and lane centers.

Our contribution is a novel algorithm based on the pixel-entropy and Dynamic Time Warping for the automatic detection of the number of lanes and their centers and the lane division lines formation, with high accuracy and low computational time. The advantages, based on the state of the art, are the following: (1) lower computational time than trajectory-based methods for the lane centers detection (converges in just 32 s with a traffic flow of one vehicle/s per-lane); (2) it is not limited by lane markings visibility, as, instead of detecting lane markings, it performs the formation of lane division lines; (3) automatic detection of the number of lanes; (4) the performance under traffic congestion is higher than trajectory-based methods; (5) automatic selection of lanes; (6) robustness to partial occlusion and shadows; and (7) no prior camera parameters initialization is needed.

## 3. Background

### 3.1. Probability Concepts

#### 3.1.1. Probability Space

A probability space [15] is a mathematical triplet (Ω,F,P) which models a random process, where Ω is the sample space, F the event space, and *P* a probability function which associates to each event $\zeta_{i}$∈F a probability $p_{i}$.

#### 3.1.2. Shannon Entropy

Let *X* be a random variable (r.v.), which can take values of a finite set, i.e., $X=\{x_{1},x_{2},\ldots ,x_{N}\}$ of cardinality *N*, with a probability distribution $P,\;p_{i}:=P\left(X=x_{i}\right)$; then, the associated Shannon entropy S(X) of a r.v. *X* is defined as follows [16]:

(1) $$S\left(X\right)=-\sum_{i=1}^{N}p_{i}\;\log\left(p_{i}\right)$$

S(X) has a maximum in the case of *equiprobability*, i.e., $p_{i}=\frac{1}{N},\;\forall i$:

(2) $$S_{max}\left(X\right)=\log\left(N\right)$$

#### 3.1.3. Tsallis Entropy

Tsallis [17] proposed a generalization of the celebrated Boltzmann–Gibbs entropy $S^{BG}$ measure able to describe extensive and non-extensive physical systems. The Tsallis entropy for a given probability distribution $P=(p_{1},\;p_{2},\;\ldots ,\;p_{N})$ or of a random variable *X*, with a probability distribution $p_{i}=P\left(X==x_{i}\right),\;i:=1,\;2,\;\ldots ,\;N$, is defined as follows:

(3) $$S^{q}\left(X\right)\equiv S^{q}\left(P\right)=\frac{1-\sum_{i=1}^{N}p_{i}^{q}}{q-1}$$

where $N\in N^{+}$ is the total number of possible microscopic configurations of the whole system, and q∈R the entropic index that characterizes the system degree of non-extensivity.

Tsallis entropy has four important mathematical properties derived from the inclusion of the entropic index *q* (see [17]).

**Figure 1:**
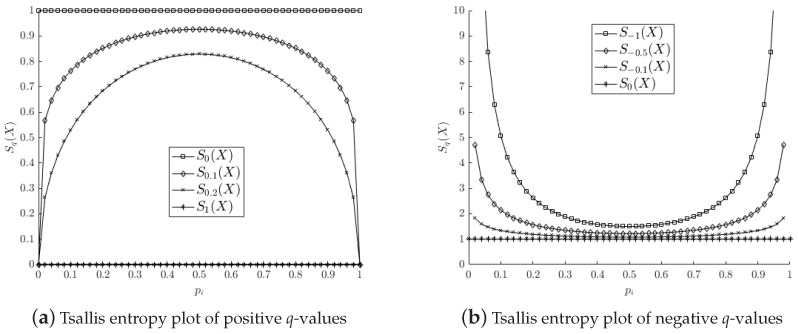
Plots of the Tsallis entropy for several entropic index values.

Figure 1 shows an illustration of the Tsallis entropy for two probabilities $p_{1}$ and $p_{2}$, where $p_{2}\;=1-p_{1}$ with several entropic index values.

#### 3.1.4. Time Series

A time series [18] is a sequence of observations on variables indexed by a set of time t. Each element of a time series is represented by the pair $\left(a_{i},\;t_{i}\right)$, where $a_{i}\in R^{n}$ is the measured value and $t_{i}$ is the associated time index. Formally, a time series *A* is described as follows:

(4) $$A=\left\{\left(a_{1},t_{1}\right),\left(a_{2},t_{2}\right),\ldots ,\left(a_{n},t_{n}\right)\right\}\;\mathrm{iff}\;\forall i,j:\left(a_{i},t_{i}\right),\left(a_{j},t_{j}\right)\in A\wedge i\leq j\Rightarrow t_{i}\leq t_{j}$$

#### 3.1.5. Histogram

A histogram H(X) is an estimator of the Probability Density Function (pdf) of a continuous random variable *X*, which can be expressed as Equation (5).

(5) $$H\left(X\right)=\left\{h_{1},\;h_{2},\;\ldots ,\;h_{m}\right\}$$

where $h_{i}=\frac{N_{i}}{N}$ is the relative frequency of *X*, *m* the number of classes known as bin, $N_{i}$ the number of observations in the class *i*, and *N* the total number of observations.

### 3.2. Peaks and Valleys

Let f(x) be a function which transforms *x* from a domain U⊆R to the domain R. A peak is defined as a local maxima of f(x), and similarly, a valley is defined as a local minima of f(x) [19]. Equations (6) and (7) define formally a peak and valley, respectively.

(6) $$f\left(x_{0}\right)\geq f\left(x\right),\;\forall x\in I$$

(7) $$f\left(x_{0}\right)\leq f\left(x\right),\;\forall x\in I$$

where $I=(x_{a},x_{b})$ is an interval such that I∩U≠∅, and $x_{0}\in I$ the peak or valley location.

**Figure 2:**
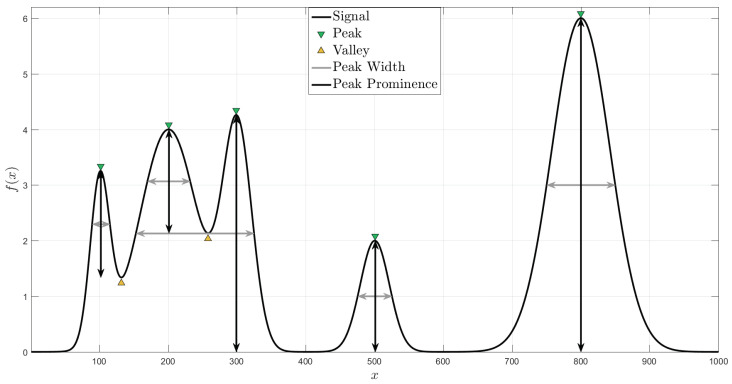
Peaks and valleys.

The peak width and prominence are two relevant features of a peak (see Figure 2). The peak width is defined as the distance between the points to the left and right of the peak where f(x) intercepts a reference height. While the prominence of a peak is the minimum vertical distance that f(x) descends before climbing back to a higher level or reaches a valley.

### 3.3. Algorithms

In this section, a short description of the algorithms K-means and DTW are presented.

#### 3.3.1. K-Means

Let *Y* be a set of observations $\left\{y_{1},\;y_{2},\;\ldots ,\;y_{N}\right\},\;y_{i}\in R^{n}$, the k-means algorithm [20] partitions the set *Y* into *k* subsets $\Upsilon =\{\Upsilon_{1},\Upsilon_{2},\ldots ,\Upsilon_{k}\},\;k<N$, such that the squares sum within each subset will be minimized in accordance with Equation (8), where $\mu_{i}$ is the mean of $\Upsilon_{i}$.

(8) $$\underset{\Upsilon }{arg min}\sum_{i=1}^{k}\sum_{y_{j}\in \Upsilon_{i}}{∥y_{j}-\mu_{i}∥}^{2}$$

#### 3.3.2. Dynamic Time Warping

Sakoe and Chiba [5] introduced the DTW algorithm to align temporal sequences, which has been widely used mainly on speech recognition [5] and time series classification [21]. Consider two time series $A=(a_{1},\;a_{2},\;\ldots ,\;a_{M})$ and $B=(b_{1},\;b_{2},\ldots ,\;b_{N})$ of length *M* and *N*, respectively, where $a_{i},b_{i}\in R^{n}$, and let *c* be a local cost function defined as a distance *d*, such that a cost matrix C of size M×N is formed, where each element $C_{i,j}$ is defined as Equation (9).

(9) $$C_{i,j}=c\left(i,j\right)=d\left(a_{i},b_{j}\right)=\|a_{i}-b_{j}\|.$$

A path *P* can be seen as an ordered set of points in $R^{n}$. A warping path $\gamma =\{\gamma_{1},\gamma_{2},\ldots ,\gamma_{K}\}$ is a sequence of tuples $\gamma_{k}=\left(i\left(k\right),j\left(k\right)\right)$ with an associated cost c(k)=c(i(k),j(k)), where *i* and *j* are the corresponding indexes of the $a_{i}$ and $b_{j}$ with its distance $C_{i,j}$, and k∈[1:K],K≥max(M,N), if and only if the following conditions are satisfied:

1. Boundary: $\gamma_{1}=\left(i\left(1\right),j\left(1\right)\right)$ and $\gamma_{K}=\left(i\left(M\right),j\left(N\right)\right)$.
2. Monotonicity: The set of indexes i(k) and j(k) of *A* and *B* are monotonically nondecreasing, i.e., i(1)≤i(2)≤…≤i(K) and j(1)≤j(2)≤…≤j(K).
3. Continuity: Consecutive nodes of γ must be reached by horizontal, vertical or diagonal steps of length 1:
  (10) $$c\left(k-1\right)=\left\{\begin{array}{c} \left(i(k),j(k)-1\right)\\ \left(i(k)-1,j(k)-1\right)\\ \left(i(k)-1,j(k)\right) \end{array}\right.$$

Then, the total cost for a warping path is given by:

(11) $$c_{\gamma }\left(A,B\right)=\sum_{k=1}^{K}c\left(a_{i(k)},b_{j(k)}\right)$$

For the series *A* and *B*, we have several warping paths γ, where each of them has a total cost $c_{\gamma }$, and the minimal ${\hat{c}}_{\gamma }$ is called distance DTW(A,B).

**Figure 3:**
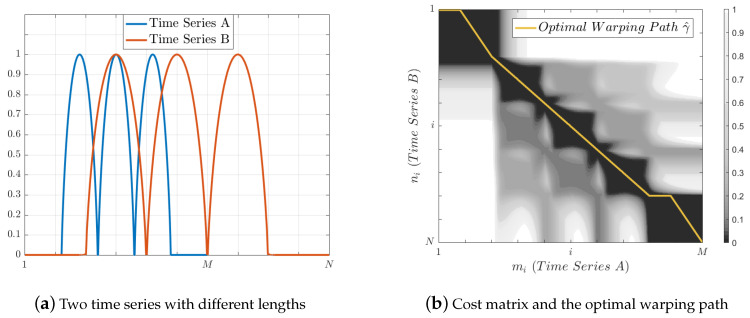

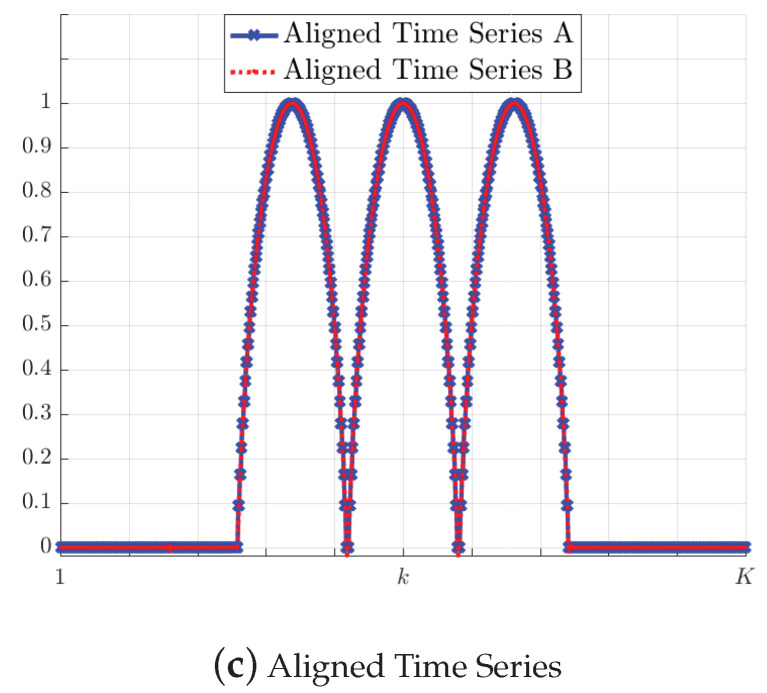
Alignment through DTW algorithm.

Let Γ be the set of all possible warping paths between *A* and *B*. An optimal warping path $\hat{\gamma }$ is defined as the path γ∈Γ with the minimum cost or distance DTW(A,B) (Equation (12)). The optimal warping path can be seen as a function of alignment whose *domain* is the set {1,2,…,K} and the *codomain* the set of pairs (i(k),j(k)). Then, it is said that the time series *A* and *B* are aligned (see Figure 3). Figure 3 shows two time series not aligned in time, Figure 3 presents its associated cost matrix and the optimal warping path found by DTW algorithm, and Figure 3 the alignment between series. It is important to note that, for any given element $a_{i}$ of *A*, there is at least one corresponding element $b_{j}$ in *B* which can be found in the optimal warping path, and vice versa.

(12) $$DTW\left(A,B\right)\equiv {\hat{c}}_{\gamma }\left(A,B\right)=\min_{\gamma \in \Gamma }\sum_{k=1}^{K}c\left(a_{i(k)},b_{j(k)}\right)$$

## 4. Automatic Lane Detection

Lane detection algorithms are able to extract road features such as lane centers, lane division lines as well as lane boundaries. From vehicle trajectories, lane centers can be estimated, but the estimation of each lane is biased by driving behaviors caused by factors such as topologies of road networks, lane position and shoulder width, lane deviation, the field-of-view angle, etc. [22]. Here, we define two types of lanes: *static and dynamic lanes*. Static lanes are those lanes for which their position is time-invariant or geometric lanes, while dynamic lanes are those lanes which their position is time-variant and depends on external factors as previously mentioned.

### 4.1. Lane Model

A trajectory *T* of an object *x* can be seen as a time-ordered set or the states or positions of the object, and it is represented as $T\left(x\right)=\left\{x_{1},x_{2},\ldots ,x_{m}\right\}$, where $x_{i}\in R^{n}$ contains a position sampled at time $t_{i}$, and *m* is the length of *T*. Its associated path $\gamma =\{x_{1},x_{2},\ldots ,x_{m}\}$ is only an ordered set.

**Figure 4:**
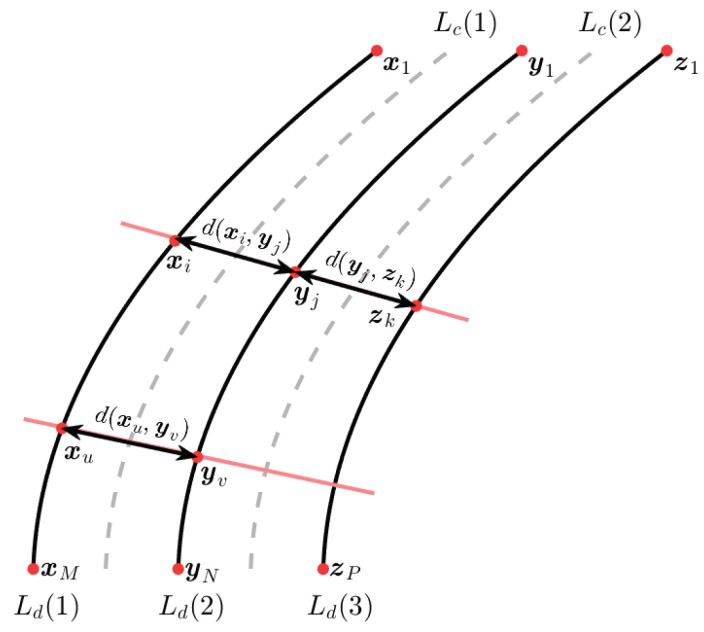
Illustration of roadway model with two lanes and three lane division lines.

A road can be modeled by *L* lanes and a set of 2L+1 paths, which represents the positions of two main features of road (see Figure 4): (i) *L* Lane Center (${\hat{L}}_{c}\left(n\right)$) is intended as a static path, whose elements are the midpoints of the lane; ND (ii) L+1 Lane Division line (${\hat{L}}_{d}\left(n\right)$) is intended as a series of markings on the road, equivalent to a path; where *n* is the lane number. Then, any lane center or lane division line L(n) can be represented by a polynomial; second or third degree polynomials describe very well several lane curvatures. For our case, we use only second-degree polynomial (see Equation (13)).

(13) $$L\left(n\right)=\{\left(x,y\right)\in R^{2}|y=p^{\left(n\right)}\left(x\right)=a_{0}+a_{1}x+a_{2}x^{2}\}$$

where *n* is the lane number, and $a_{k}$ the coefficients of the polynomial. For our models, discrete values of (x,y) points will be pixel positions denoted as $\phi_{\left(i,j\right)}^{\left(n\right)}$.

Given the curves representing the lanes as shown in Figure 4 and two different normal lines, at intersection points, the following properties hold: the Euclidean distance $de\left(x_{i},y_{j}\right)=de\left(x_{u},y_{v}\right)$ for regular lanes at *k*-th lane and $de\left(x_{i},y_{j}\right)=de\left(y_{j},z_{k}\right)$ when the two lanes have the same width.

Our goal is, given a certain number of estimated data points, to find the polynomial representing ${\hat{L}}_{c}\left(n\right)$ and ${\hat{L}}_{d}\left(n\right)$ that best fit the lanes in the sense of mean squared error.

### 4.2. The Proposed Algorithm

In this section, we introduce the proposed algorithm for automatic detection of the number of lanes, lane centers and lane division lines formation based on the pixel-entropy and DTW. Our approach must perform five main tasks: parameter initialization, pixel histogram construction and pixel-entropy calculation, peak features extraction and classification, peak matching, and lane center and lane division line model by a second-degree polynomial fitting.

**Figure 5:**
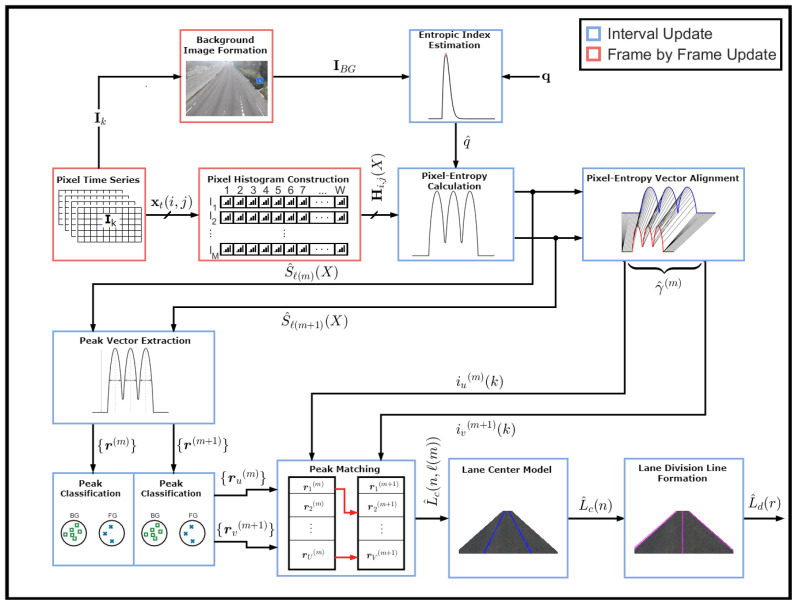
Block diagram for the lane detection algorithm.

The lane detection algorithm based on the pixel-entropy is composed of the following blocks according to the diagram illustrated in Figure 5:

1. Pixel Time Series
2. Background Image Formation
3. Entropic Index Estimation
4. Pixel Histogram Construction
5. Pixel-entropy Calculation
6. Pixel-Entropy Vector Alignment
7. Peak Features Vector Extraction
8. Peak Classification
9. Peak Matching
10. Lane Center Model
11. Lane Division Line Formation

Given a video $V_{N}$ of duration τ (N-frames) (see Figure 5), we need to calculate the pixel-entropy $S_{i,j}\left(X\right)$. Assuming that the traffic load is one vehicle/s per lane, we found that after 30 s the relative frequency corresponding to the pixel-entropy converge to a value, and the time needed for this convergence is denoted here as the transient time $t_{r}$. Besides, for each frame, the histogram of several rows of pixels will be computed and accumulated for each pixel belonging to the *j*-th row. After $t_{r}$ has been elapsed, the entropy of each pixel is calculated, forming a pixel-entropy vector or curve for each *j*-th row of pixels, which will contain local maxima and minima that determine the lanes. Then, our algorithm estimates the lane centers and lane division lanes. For updating, this process will be repeated after a certain amount of time $t_{u}$.

#### 4.2.1. Pixel Time Series

Let $V_{N}=\left\{I_{1},\;I_{2},\;\ldots ,\;I_{N}\right\}$ be a video or a sequence of *N* frames of W×H (Width×Height) pixels, where the intensity values of each pixel in the grayscale can be represented by a discrete random variable *X*, then each pixel can be modeled by a time series $x_{t}\left(i,j\right)$, where *t* is the discrete time index and (i,j) the pixel position.

#### 4.2.2. Background Image Estimation

A simple background image estimation is performed to separate moving objects in foreground from the background, which can be modeled by $I_{BG_{k}}=\left\{x_{k}\left(i,j\right)\;|\;x_{k}\left(i,j\right)\in R_{g},\;1\leq i\leq W,\;1\leq j\leq H\right\}$ which satisfies some background criterion [23], e.g., the statistical mode of each $x_{t}\left(i,j\right)$ at which the Probability Mass Function (pmf) takes its maximum.

#### 4.2.3. Pixel Histogram Construction

For each pixel, its histogram $H_{i,j}\left(X\right)=H\left(x_{t}\left(i,j\right)\right)$ is computed and updated frame by frame. Any object moving across the background will change $x_{t}\left(i,j\right)$ for all its associated pixels and almost always the histogram as well. Then, its associated pixel pmf $f_{X}\left(x\right)$ is estimated and updated (to have a fair comparison of each pixel pmf, the number of bins or classes of each histogram is fixed and equal for all). The pmf $f_{X}\left(x\right)$ is the key input to the entropy estimators (see Section 4.2.4). Besides, histograms are the basis for the background image formation (see Section 4.2.2).

#### 4.2.4. Pixel-Entropy Estimation

Based on $f_{X}\left(x\right)$, the pixel-entropy $S_{i,j}\left(X\right)$ is calculated as either Shannon or Tsallis by Equations (1) or (3), respectively. Several rows of pixels are selected; for each *j*-th row, a pixel-entropy vector $S_{j}\left(X\right)$ is formed (see Equation (14)), where each element of this vector is the pixel-entropy $S_{i,j}\left(X\right)$ at location (i,j) and *j* the selected row.

(14) $$S_{j}\left(X\right)=\left(S_{1,j}\left(X\right),S_{2,j}\left(X\right),\ldots ,S_{i,j}\left(X\right),\ldots ,S_{W,j}\left(X\right)\right)$$

where i∈[1,W] and *j* takes *M* from the *H* (height of the image) possible values.

Based on the assumption that the pixel-entropy changes over time or not, three types of theoretical pixel-entropy behaviors can be distinguished:

1. A pixel located at the center of the lane shows a high entropy value.
2. A pixel located at the lane division line shows a relative low entropy value.
3. A pixel located out of the road shows entropy values close to zero.

**Figure 6:**
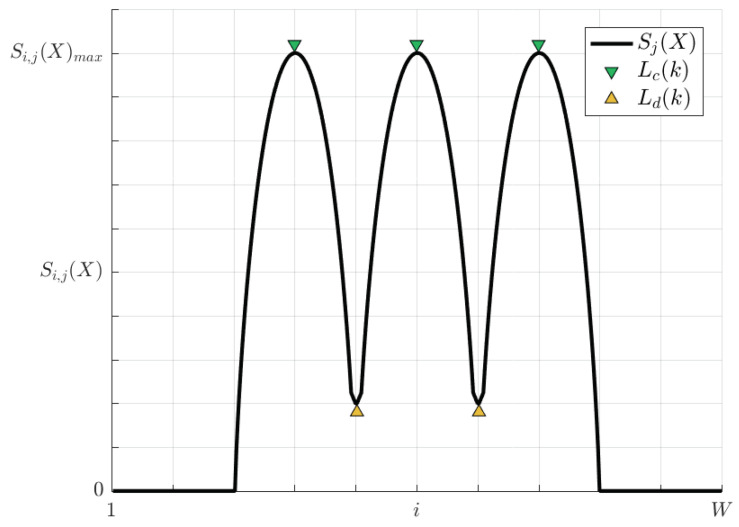
Theoretical pixel-entropy vector for a road scenario with three lanes.

Any of the *M* theoretical selected pixel-entropy vectors $S_{j}\left(X\right)$ can be modeled as Figure 6, where each lane is represented as a wave cycle, while lane centers and lane division lines are the local maxima and minima, respectively.

**Figure 7:**
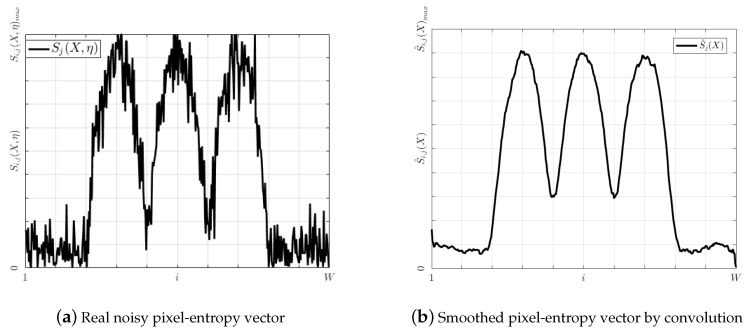
Effects of noise on the pixel-entropy vector.

During the acquisition process of an image, noises are added due to physical sensors and technology limitations [24]. As a result, pixel time series will be contaminated. Since $S_{i,j}\left(X\right)$ is calculated from a noisy pixel time series, $S_{j}\left(X\right)$ will be distorted. To take into account the noise on the pixel-entropy vector, Equation (14) can be rewritten as Equation (15), where η is a r.v. which models the noise of unknown distribution and $S_{j}\left(\eta \right)$ its associated pixel-entropy vector. A noisy pixel-entropy vector is shown in Figure 7.

(15) $$S_{j}\left(X,\eta \right)=S_{j}\left(X\right)+S_{j}\left(\eta \right)$$

To minimize the noise effects, $S_{j}\left(X,\eta \right)$ is convolved with a Moving Average (MA) filter of order *N* by Equation (16), where $h_{MA}$ is the impulse response of the MA filter. As a result of the convolution, a smoothed pixel-entropy vector ${\hat{S}}_{j}\left(X\right)$ is estimated which will have as many peaks as lanes and one more valleys than lanes. A smoothed pixel-entropy vector is shown in Figure 7.

(16) $${\hat{S}}_{j}\left(X\right)=S_{j}\left(X,\eta \right)*h_{MA}$$

#### 4.2.5. Entropic Index Estimation

Since Tsallis entropy considers a system as a non-extensive through the entropic index *q*, its estimation is crucial for entropy calculation. A procedure to estimate *q* is just well-known for particular cases [25,26], so there is not a closed-form expression to estimate the entropic index for a given system.

Ramírez-Reyes et al. [6] proposed a procedure to estimate the characteristic Tsallis entropic index *q* of an image based on the *q*-redundancy maximization for a finite set of *q* values.

We used this methodology to estimate the entropic index from a background image $I_{BG}$, estimated after a time denoted by $t_{r}$ for the associated video $V_{n}$.

#### 4.2.6. Automatic Pixel Rows Selection

**Figure 8:**
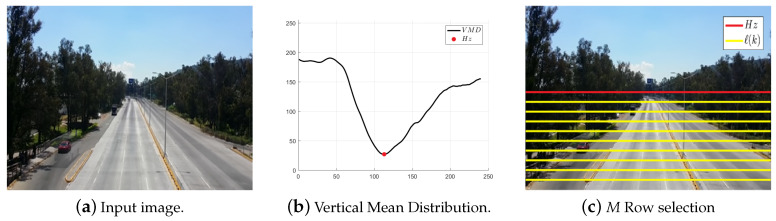
Vertical mean distribution and its local minimum for the input image.

Multimodal scenarios are a common environmental challenge for lane detection. Lu et al. [7] addressed this issue by detecting the horizon line (Hz) where the lane boundaries converge in a point referred to in the literature as the vanishing point of the road ${VP}_{road}$, which partitions a W×H image into sky and road regions. Horizon line is calculated by searching for the first minimum value along the Vertical Mean Distribution (VMD) of an image [7]. First, VMD is computed by averaging the gray value of each row on the image. Subsequently, a local minimum searching algorithm is performed to find the location of the most prominent local minimum value on the VMD, where this location corresponds with Hz (see Figure 8).

To process only rows of pixels located inside of the road, horizon line Hz is computed, such that the number of rows required to represent each lane center or lane division line will be H−Hz, which leads to an expensive memory footprint cost. To reduce the memory required to store the H−Hz pixel-entropy vectors and to improve the algorithm performance, only *M* of the H−Hz possible rows are selected. Then, *M* full rows of pixels are equally spaced by steps of $\frac{H-Hz}{M}$, where each of these rows are indexed by:

(17) $$\ell \left(k\right)=Hz+\left(k-1\right)\frac{H-Hz}{M},\;\left|\;k=1,2,\ldots ,M\;\right|\;Hz\in \left[1,H\right]$$

#### 4.2.7. Peak Vectors Extraction

Any theoretical pixel (see Figure 6) containing an entropy peak can be represented as a vector r=(loc,pk,w,pr), where $loc\in R^{2}$ is its location, pk is the pixel-entropy value, *w* is the peak width and pr is the peak prominence. Then, for each of the selected *M* rows, we have at least as many peaks vectors as number of lanes inside of the road, and other peaks outside it, which are extracted.

#### 4.2.8. Peak Classification Filtering

**Figure 9:**
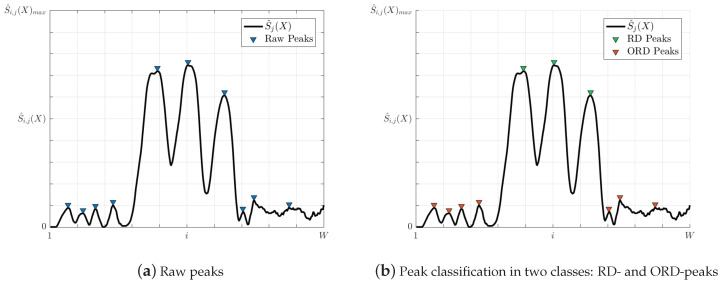
Illustration of the peak classification filtering.

As shown in Figure 9, not only the local maxima corresponding to the lane centers appear, but also other additional peaks can be observed: inside outside the road. One way to separate these peaks is by classification. Jacobson [27] addressed the problem of the classification of peaks by calculating the thresholds of two types of peaks. In this paper, this algorithm is implemented for the same purpose, where the classes are labeled as on road peak (RD) if their location is within the road, or as out of road peak (ORD) if their location is outside of the road. Finally, the background peaks are filtered out (see Figure 9).

#### 4.2.9. Peak Matching Algorithm

At this point, we have:

1. A Region of Interest delimited by the road horizon line and the bottom line of the image;
2. *M* selected rows, which are the basis for the lane detection; and
3. The set of all on-road peak-vectors.

The goal of this algorithm is to select all points belonging to each lane center. Then, we need to find the lane centers, the lane division lines and the number of lanes of the road.

**Figure 10:**
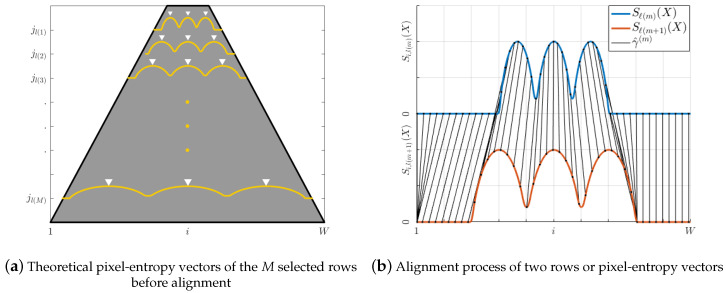
Pixel-entropy vectors alignment.

It is a well-known fact that, for image analysis, the intensity values of the pixels have long-range correlations [28]. Consequently, two consecutive pixel-entropy vectors $S_{j}\left(X\right)$ and $S_{j+1}\left(X\right)$ are highly correlated with each other for j=1,2,…,H (image height). Thus, for the *M* selected rows, $S_{l(k+1)}\left(X\right)$ is an approximated scaled version of $S_{l(k)}\left(X\right)$. Besides, each peak vector at row ℓ(m) does not have the same characteristics of its associated peak vector at row ℓ(m+1), as Figure 10 shows, hence an algorithm for pixel-entropy vector alignment and peak matching is required (see Figure 10).

**Figure 11:**
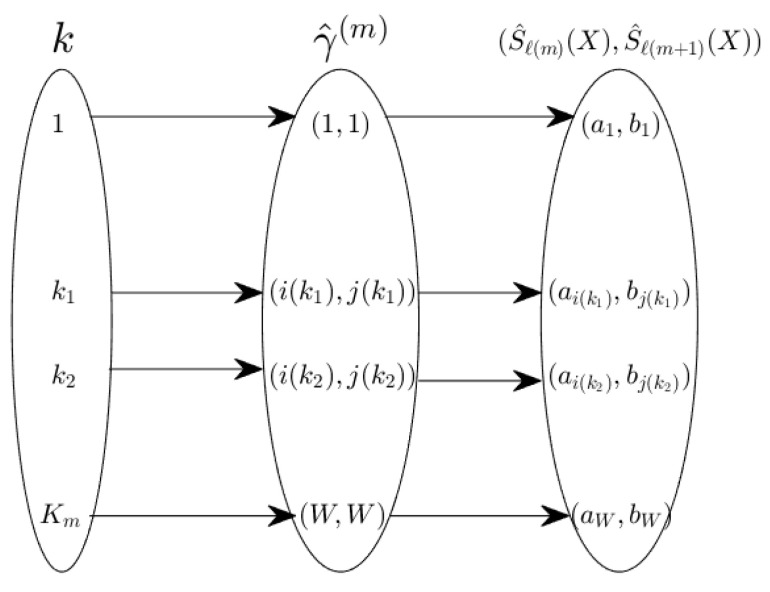
*k*-domain, codomain and indexing of ${\hat{S}}_{\ell (X)}$ for the optimal warping path ${\hat{\gamma }}^{\left(m\right)}$.

**Figure 12:**
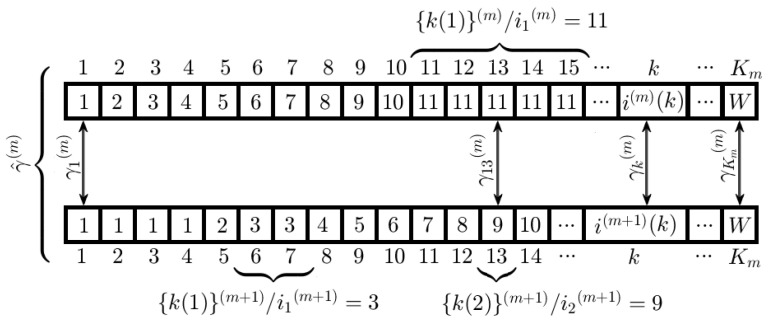
An example of the structure for the optimal warping path ${\hat{\gamma }}^{\left(m\right)}$.

**Figure 13:**
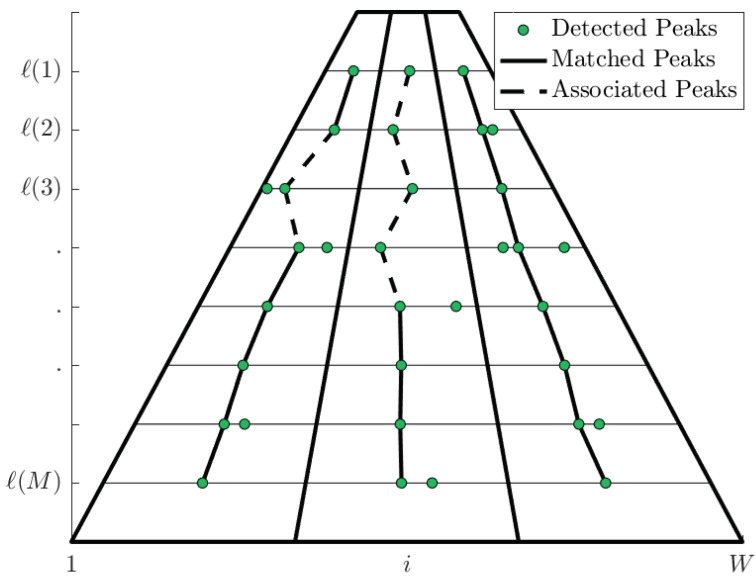
Peak matching example.

We implement an algorithm for peak matching based on DTW. The steps of the peak matching algorithm are the following:

1. The pair of rows or pixel-entropy vectors ${\hat{S}}_{\ell (m)}\left(X\right)$ and ${\hat{S}}_{\ell (m+1)}\left(X\right)$ are aligned by the DTW algorithm (see Section 3.3.2). As a result of the alignment, the optimal warping path ${\hat{\gamma }}^{\left(m\right)}$ and the distance $DTW({\hat{S}}_{\ell (m)}\left(X\right),{\hat{S}}_{\ell (m+1)}\left(X\right))$ at rows ℓ(m) and ℓ(m+1) are computed, where ${\hat{\gamma }}^{\left(m\right)}$ is the set of tuples $\left\{{\gamma_{1}}^{\left(m\right)},{\gamma_{2}}^{\left(m\right)},\ldots ,{\gamma_{k}}^{\left(m\right)},\ldots ,{\gamma_{K_{m}}}^{\left(m\right)}\right\}$ whose domain is $1\leq k\leq K_{m}$ and $K_{m}\geq W$. Each tuple ${\gamma_{k}}^{\left(m\right)}=\left(i^{\left(m\right)}\left(k\right),i^{\left(m+1\right)}\left(k\right)\right)$ is an ordered pair of indexes that refers to a pair of elements $\left({\hat{S}}_{i^{\left(m\right)}\left(k\right),\ell \left(m\right)},{\hat{S}}_{i^{\left(m+1\right)}\left(k\right),\ell \left(m+1\right)}\right)$, as Figure 11 shows, where each $\left(a_{i^{\left(m\right)}\left(k\right)},b_{i^{\left(m+1\right)}\left(k\right)}\right)$ is the pair of elements. This is an alignment process.
2. For the ℓ(m) and ℓ(m+1) rows, a set of on-road entropy-peaks are detected and extracted. Each entropy-peak is associated with a feature vector ${r_{u}}^{\left(m\right)}=\left(i^{\left(m\right)}\left(k\right),\ell \left(m\right),{\hat{S}}_{i^{\left(m\right)}\left(k\right),\ell \left(m\right)}\left(X\right),w_{u},p_{u}\right)$, where $w_{u}$ and $p_{u}$ are geometrically extracted (see Section 4.2.7). Let *U* be the number of on-road entropy-peaks associated to the peak-vectors ${r_{1}}^{\left(m\right)},{r_{2}}^{\left(m\right)},\ldots ,{r_{U}}^{\left(m\right)}$ at row ℓ(m), and *V* be the number of on-road entropy-peaks associated to the peak-vectors ${r_{1}}^{\left(m+1\right)},{r_{2}}^{\left(m+1\right)},\ldots ,{r_{V}}^{\left(m+1\right)}$ at row ℓ(m+1), then:
  (a) **Ideal case**: U=V=L, such that ${\hat{S}}_{\ell (m)}\left(X\right)$ has *L* on-road entropy-peaks, as Figure 6 shows.
  (b) **Real case**: U≠V, in practice more than *L* entropy-peaks can be detected on the pixel-entropy vector ${\hat{S}}_{\ell (m)}\left(X\right)$ due to the distortion introduced by the noise (see Figure 7).
3. Once a pair of rows have been aligned, we work only with those points of the domain of the optimal warping path ${\hat{\gamma }}^{\left(m\right)}$ (called here *k*-domain) and the corresponding pair of indexes of its codomain, where live the *U* on-road peak-vectors of $S_{\ell (m)}\left(X\right)$ and *V* on-road peak-vectors of $S_{\ell (m+1)}\left(X\right)$. It is very important to note that the inverse of this functions is not a function but a relation, i.e., to each of the *U* or *V* peak-vectors corresponds one or more *k* values, denoted as k(u) and k(v), respectively, that together build two sets ${\left\{k\left(u\right)\right\}}^{\left(m\right)}$, ${\left\{k\left(v\right)\right\}}^{\left(m+1\right)}$ of cardinality $N_{u}$ and $N_{v}$, respectively (see Figure 12). In Figure 12, for the peak-vectors ${r_{1}}^{\left(m\right)}$ and ${r_{1}}^{\left(m+1\right)}$, their subsets in the *k*-domain are ${\left\{k\left(1\right)\right\}}^{\left(m\right)}=\left\{11,12,13,14,15\right\}$, ${\left\{k\left(1\right)\right\}}^{\left(m+1\right)}=\left\{6,7\right\}$ and their corresponding locations ${i_{1}}^{\left(m\right)}=11$, ${i_{1}}^{\left(m+1\right)}=3$. Then, there are not points in the *k*-domain referencing via ${\gamma_{k}}^{\left(m\right)}=\left({i_{1}}^{\left(m\right)},{i_{1}}^{\left(m+1\right)}\right)$ to ${r_{1}}^{\left(m\right)}$ and ${r_{1}}^{\left(m+1\right)}$ simultaneously. However, for the vectors ${r_{1}}^{\left(m\right)}$ and ${r_{2}}^{\left(m+1\right)}$, ${\left\{k_{2}\right\}}^{\left(m+1\right)}=13$ and for k=13, ${\gamma_{13}}^{\left(m\right)}=\left(11,9\right)$ that relates to ${r_{1}}^{\left(m\right)}$ and ${r_{2}}^{\left(m+1\right)}$.
4. The associated vector is ${r_{u}}^{\left(m\right)}=\left(u,\ell \left(m\right),S_{u,\ell (m)}\left(X\right),w_{u},p_{u}\right)$, u=i(ℓ(m),k), where (u,ℓ(m)) is the location in the background image of a pixel that probably belongs to the *l*-th lane center or ${\hat{\phi }}_{u,\ell (m)}^{n}$ and the pair of pixels $\left({\hat{\phi }}_{u,\ell (m)}^{n},{\hat{\phi }}_{v,\ell (m+1)}^{n}\right)$ builds a possible segment of the lane center $L_{c}\left(l\right)$. Then, given two peak-vectors ${r_{u}}^{\left(m\right)}$ and ${r_{v}}^{\left(m+1\right)}$, we say that $r_{u}$ is related with $r_{v}$, ${r_{u}}^{\left(m\right)}\;R\;{r_{v}}^{\left(m+1\right)}$, by the minimal distance $d({r_{u}}^{\left(m\right)},{r_{v}}^{\left(m+1\right)})$ in the *k*-domain defined as:
  (18) $$d\left({r_{u}}^{\left(m\right)},{r_{v}}^{\left(m+1\right)}\right)=\left|k\left(u\right)-k\left(v\right)\right|$$
  It is obvious that $d({r_{u}}^{\left(m\right)},{r_{v}}^{\left(m+1\right)})=0$, iff $\exists !k\in \;k-\mathrm{domain}\;:{\gamma_{k}}^{\left(m\right)}=\left({i_{u}}^{\left(m\right)},{i_{v}}^{\left(m+1\right)}\right)$, then it is said that ${r_{u}}^{\left(m\right)}$ and ${r_{v}}^{\left(m+1\right)}$ are matched, and, in other cases, are associated.
  In the **ideal case**, $\forall {r_{u}}^{\left(m\right)}\in U\;\mathrm{and}\;{r_{v}}^{\left(m+1\right)}\in V,d\left({r_{u}}^{\left(m\right)},{r_{v}}^{\left(m+1\right)}\right)=0$, there is a one-to-one correspondence between the *U* and *V* peak-vectors. However, for the **real case**, the correspondence of the *U* and *V* peak-vectors is not always one-to-one, that is, sometimes they are matched, and sometimes they are associated only (see Figure 12).
5. For each *m*-value, m=1,2,…,M−1, all possible relations between pairs of peak-vectors $\left({r_{u}}^{\left(m\right)},{r_{v}}^{\left(m+1\right)}\right)$ can be represented by a cost matrix ξ of size U×V (Equation (20)), where each element $\xi_{u,v}=d\left({r_{u}}^{\left(m\right)},{r_{v}}^{\left(m+1\right)}\right)$ is calculated by Equation (18). The pairs of on-road peak-vectors are selected according to the following rules:
  (a) For each *v*-column of ξ, a peak-vector ${r_{v}}^{\left(m+1\right)}$ is matched or associated with a peak-vector ${r_{u}}^{\left(m\right)}$, if its distance is minimal for the whole column.
  (b) Finally, if there are two or more peak-vectors in row ℓ(m) or ℓ(m+1) that are related to the same peak-vector in the other row, i.e., ℓ(m+1) or ℓ(m), our algorithm selects the one with the greatest peak width, so noisy peaks will be filtered automatically.
  For each *j*:
  (19) $$\underset{1\leq i\leq U}{arg min}\xi_{ij}:=\left\{i|i\in \left[1,U\right]\;\mathrm{and}\;\forall k\in \left[1,V\right],\xi_{kj}\geq \xi_{ij}\right\}$$
  In general, for each *m*-value ξ has the form:
  (20) $$\begin{array}{c} \xi =\left[\begin{array}{ccc} \xi_{11} & \cdots & \xi_{1V}\\ \vdots & \ddots & \vdots \\ \xi_{U1} & \cdots & \xi_{UV} \end{array}\right] \end{array}$$
  (a) **Ideal case**: The size of ξ is L×L, with zeros on the main diagonal and values corresponding to multiples of the lane width $L_{w}$ elsewhere as Equation (21).
    (21) $$\begin{array}{c} \xi =\left[\begin{array}{ccc} \xi_{11} & \cdots & \xi_{1L}\\ \vdots & \ddots & \vdots \\ \xi_{L1} & \cdots & \xi_{LL} \end{array}\right]=\left[\begin{array}{ccccc} 0 & L_{w} & 2L_{w} & \cdots & \left(L-1\right)L_{w}\\ L_{w} & 0 & L_{w} & \cdots & \left(L-2\right)L_{w}\\ 2L_{w} & L_{w} & 0 & \cdots & \left(L-3\right)L_{w}\\ \vdots & \vdots & \vdots & \ddots & \vdots \\ \left(L-1\right)L_{w} & \cdots & 2L_{w} & L_{w} & 0 \end{array}\right] \end{array}$$
  (b) **Real case**: The size of ξ is U×V, the distances $d({r_{u}}^{\left(m\right)},{r_{v}}^{\left(m+1\right)})$ are calculated and the selection rules 5a) and 5b) are applied.
    An example of a cost matrix between the rows ℓ(M−1) and ℓ(M) of Figure 13 is shown in Equation (22), with U=5 and V=4, where the pairs of peak-vectors that probably belong to the lane center are ${r_{1}}^{\left(M-1\right)}\;R\;{r_{1}}^{\left(M\right)}$, ${r_{3}}^{\left(M-1\right)}\;R\;{r_{2}}^{\left(M\right)}$, ${r_{3}}^{\left(M-1\right)}\;R\;{r_{3}}^{\left(M\right)}$, ${r_{4}}^{\left(M-1\right)}\;R\;{r_{4}}^{\left(M\right)}$, and where the peak-vectors ${r_{2}}^{\left(M-1\right)}$ and ${r_{5}}^{\left(M-1\right)}$ are discarded because there is no a minimum distance in their columns. Besides, as ${r_{2}}^{\left(M\right)}$ and ${r_{3}}^{\left(M\right)}$ are related with the same vector ${r_{3}}^{\left(M-1\right)}$, the peak-vector with the smallest peak width is discarded, and this is ${r_{3}}^{\left(M\right)}$. Finally, each pair of the selected peak-vectors are $\left({r_{1}}^{\left(M-1\right)},{r_{1}}^{\left(M\right)}\right)$, $\left({r_{3}}^{\left(M-1\right)},{r_{2}}^{\left(M\right)}\right)$ and $\left({r_{4}}^{\left(M-1\right)},{r_{4}}^{\left(M\right)}\right)$.
    (22) ξ=0100110200109010019010001010020010090021011010010
6. For each *m* value indexing $\left(S_{\ell (m)}\left(X\right),S_{\ell (m+1)}\left(X\right)\right)$, each selected pair of peak-vectors is stored in a path ${\hat{L}}_{c}\left(n,\ell \left(m\right)\right)$ (see Equation (23)), which is updated for the next m=m+1 repeating Steps 1–6, and the estimated lane center is given by the set of the selected pairs of peak-vectors.
  (23) $${\hat{L}}_{c}\left(n,\ell \left(m\right)\right)=\left\{\left({r_{i}}^{\left(m\right)},{r_{j}}^{\left(m+1\right)}\right),\left({r_{j}}^{\left(m+1\right)},{r_{p}}^{\left(m+2\right)}\right)\right\}$$
  or by the ordered set of the corresponding locations:
  (24) $${\hat{L}}_{c}\left(n,\ell \left(m\right)\right)=\left\{{\hat{\phi }}_{u,\ell (m)}^{n},{\hat{\phi }}_{v,\ell (m+1)}^{n},\ldots ,{\hat{\phi }}_{z,\ell (M)}^{n}\right\}$$
  where *n* is the lane center number, and where ${\hat{\phi }}_{i,j}^{n}$ represents the estimated location of the pixel (i,j) in the background image, belonging to the *n*-th lane.
  Consequently:
  (a) The number of paths is the number of dynamic lane centers found and, therefore, the number of lanes. Note that the dynamic lane centers are the real lane centers, while the static lane centers are those virtual paths built geometrically with de midpoints of the lane division lines.
  (b) The estimated lane center $L_{c}$ is given by Equation (24).
  (c) The length of each lane ${\hat{L}}_{c}\left(n,\ell \left(m\right)\right)$ is given by the sum of the lengths of each segment determined by $\left({r_{i}}^{\left(m\right)},{r_{j}}^{\left(m+1\right)}\right)$.

**Figure 14:**
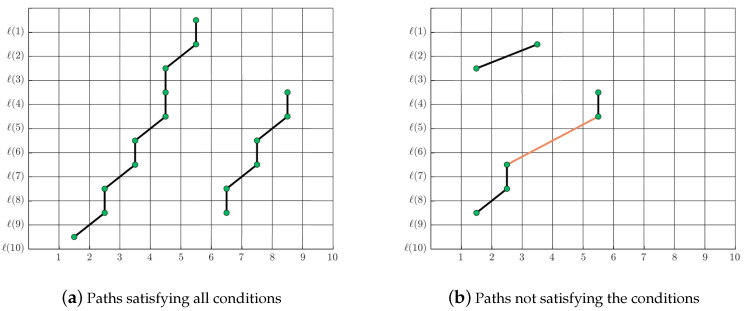
Illustration of paths conditions with M=10 and W=10.

Each path $L_{c}\left(n,\ell \left(m\right)\right)$ must satisfy the following conditions (see Figure 14):

1. A path must contain at least a certain minimum number of points, e.g., 3 in our case.
2. A path does not have a gap, and each path must be continuous.

Therefore, the described algorithm allows obtaining automatically not only the lane center paths, but also the number of lanes of any road.

#### 4.2.10. Lane Center Model

Each path ${\hat{L}}_{c}\left(n,\ell \left(m\right)\right)=\left\{{\hat{\phi }}_{u,\ell (m)}^{\left(n\right)},{\hat{\phi }}_{v,\ell (m+1)}^{\left(n\right)},\ldots ,{\hat{\phi }}_{z,\ell (M)}^{\left(n\right)}\right\}$ is fitted by a second degree polynomial using $L\left(n\right)=\{\left(x,y\right)\in R^{2}|y=p^{\left(n\right)}\left(x\right)=a_{0}+a_{1}x+a_{2}x^{2}\}$ to model a lane center as a path ${\hat{L}}_{c}\left(n\right)$, where *n* indexes the lane number.

#### 4.2.11. Lane Division Lines Formation

A road with *L* lanes has: L−1 internal lane division lines, and left and right lane division lines. Besides, without loss of generality, for the lane centers, Equation (24) as an ordered set can be rewritten for each *n*-th lane by:

(25) $${\hat{L}}_{c}\left(n,\ell \left(m\right)\right)=\left\{{\hat{\phi }}_{1,\ell (1)}^{\left(n\right)},{\hat{\phi }}_{2,\ell (2)}^{\left(n\right)},\ldots ,{\hat{\phi }}_{k,\ell (m)}^{\left(n\right)},\ldots ,{\hat{\phi }}_{M,\ell (M)}^{\left(n\right)}\right\}$$

where *k* indicates the order of the points in the path, m=1,2,…,M indexes the selected rows ℓ(m), and n=1,2,…,L indexes the lane number.

**Figure 15:**
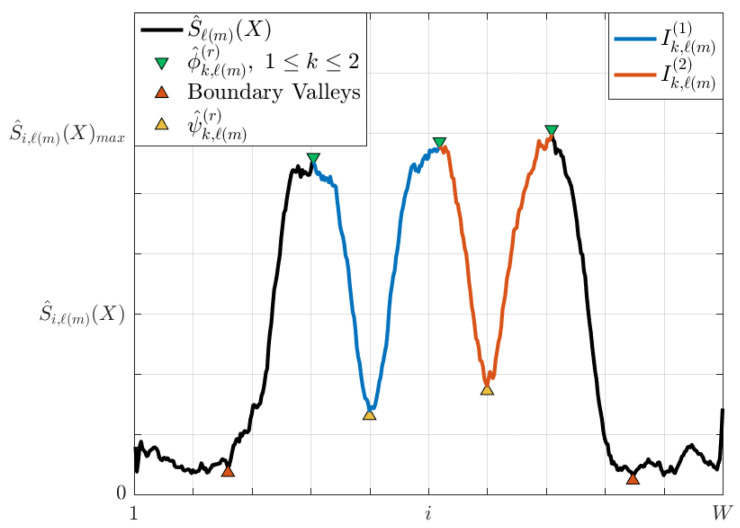
Lane division lines formation.

Lane division lines are formed by finding, for each of the *M* selected rows, the pixel with the minimum entropy value in the interval defined by the two peak-vectors that belong to the adjacent lane center paths. Then, given ${\hat{L}}_{c}\left(n,l\left(m\right)\right)$, any lane division line ${\hat{L}}_{d}\left(r,l\left(m\right)\right)$ is expressed as:

(26) $${\hat{L}}_{d}\left(r,\ell \left(m\right)\right)=\left\{{\hat{\psi }}_{1,\ell (1)}^{\left(r\right)},{\hat{\psi }}_{2,\ell (2)}^{\left(r\right)},\ldots ,{\hat{\psi }}_{k,\ell (m)}^{\left(r\right)},\ldots ,{\hat{\psi }}_{M,\ell (M)}^{\left(r\right)}\right\}$$

where r=0,1,2,…,L indexes the lane division line number and where each ${\hat{\psi }}_{k,\ell (m)}^{\left(r\right)}$ is found according to the following rules (see Figure 15):

1. For internal lane division lines 1≤r≤L−1: for each *r* and m=1,2,…,M, there are *M* intervals $I_{k,\ell (m)}^{\left(r\right)}=\left({\hat{\phi }}_{k,\ell (m)}^{\left(r\right)},{\hat{\phi }}_{k,\ell (m)}^{\left(r+1\right)}\right))$ such that each of these intervals has a point ${\hat{\psi }}_{k,\ell (m)}^{\left(r\right)})\in I_{k,\ell (m)}^{\left(r\right)}$ with the lowest entropy $min\;{\hat{S}}_{\ell (m)}\left(X\right)$, therefore ${\hat{\psi }}_{k,\ell (m)}^{\left(r\right)})\in {\hat{L}}_{d}\left(r,\ell \left(m\right)\right)$ and ${\hat{L}}_{d}\left(r,\ell \left(m\right)\right)$ is formed with the *M* points ${\hat{\psi }}_{k,\ell (m)}^{\left(r\right)})$ of the *r*-th lane.
2. As lane boundary lines do not have any lane center reference on leftmost and rightmost, the nearest boundary valley is used as a reference. For the left lane division line (r=0), ${\hat{\psi }}_{k,\ell (m)}^{\left(0\right)}$ are the nearest boundary valleys at the left side, while, for the right lane division line (r=L), ${\hat{\psi }}_{k,\ell (m)}^{\left(L\right)}$ are the nearest boundary valleys at the right side.

Each path ${\hat{L}}_{d}\left(r,\ell \left(m\right)\right)$ is fitted by a second-degree polynomial using $L\left(n\right)=\{\left(x,y\right)\in R^{2}|y=p^{\left(n\right)}\left(x\right)=a_{0}+a_{1}x+a_{2}x^{2}\}$ to model a lane division line as a path ${\hat{L}}_{d}\left(r\right)$.

#### 4.2.12. Practical and potential use and importance of this algorithm

##### Practical use

1. Instead of static or rigid geometrical lanes, dynamic lanes are detected, which are richer than static ones.
2. Dynamic lanes can capture real behaviors of the vehicles such as temporal reduction of lanes number due to: accidents, traffic jam, road maintenance, etc.
3. Layout of the lane division lines can be corrected.

##### Potential traffic applications

1. As the region of interest for this issue of vehicle traffic is found automatically, this part of the algorithm can be integrated in surveillance systems for vehicle traffic.
2. V2I and V2V can be used to communicate real time alerts and warnings related to deviations of dynamic lanes from static lanes, and other events related to local traffic.

##### Importance of the algorithm for other approaches

Whenever entropy variations can be mapped or correspond to certain regions of interest, the use of pixel entropy concept can be used as basis for the development of new algorithms, e.g., monitoring of certain activities for video applications.

## 5. Experiments and Results

### 5.1. Test Videos and Environment

**Figure 16:**
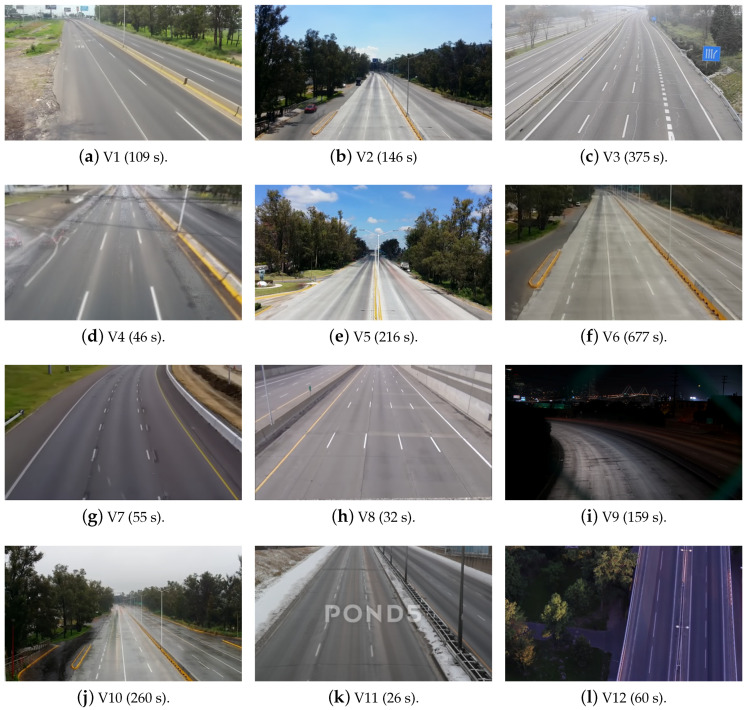
Background extracted from the test videos.

To evaluate the performance of the proposed algorithm, a large dataset consisting of 12 surveillance videos with different challenging scenarios such as image illumination, camera settings, traffic load per-lane and with other moving objects such as pedestrian and waving vegetation were used. The set of test videos have more than 2340 s of traffic scenes previously recorded from a surveillance camera and a smart phone with a resolution of 420×240 pixels and a frame rate of 25 frames per second (FPS). Figure 16 shows the background images of the test videos previously extracted, while Table 1 summarizes relevant technical data of the test videos.

The lane detection algorithm was implemented in Matlab running on a dual core 2.4GHz intel core i5 machine with 8GB of RAM. The transition time $t_{r}$ was fixed empirically to 30 s based on the assumption that the traffic load is 1 vehicle/s per-lane, while the update time $t_{u}$ to 1 s for a fast lane update. Finally, the number of bins of each histogram was fixed to 20 while the moving average filter length to 10. The lane centers and lane division lines were extracted geometrically based on the pavement markings. Consequently, they do not show some of the drivers’ dynamic behaviors.

### 5.2. Pixel-Entropy Measurements

**Figure 17:**
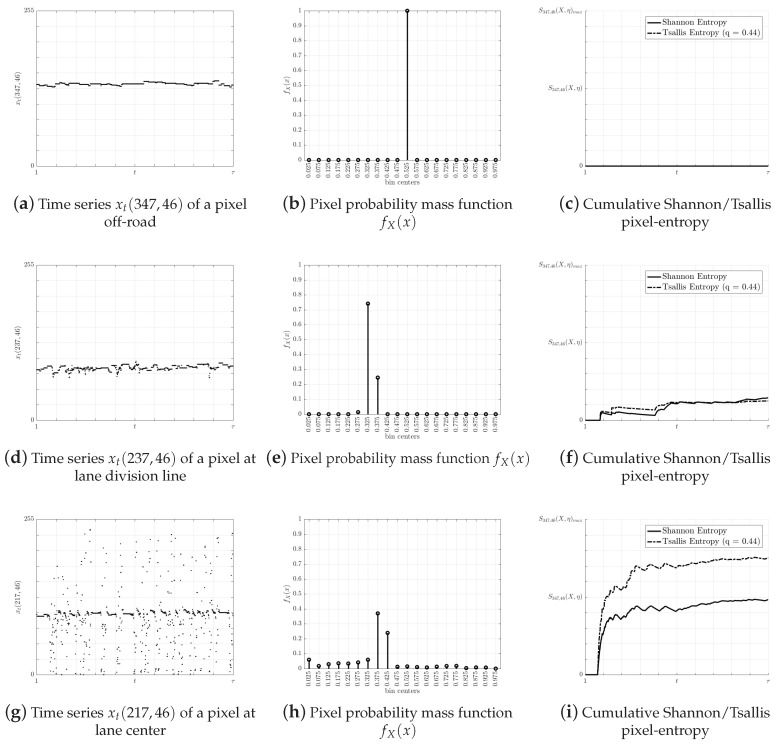
Pixel-entropy behavior.

To validate the assumptions about the pixel-entropy behavior, a set of measurements were taken. First, three pixels time series from video V7 were extracted. Next, their histograms were computed with nbin=20, following by the calculation of the cumulative Shannon and Tsallis entropies and the entropic index $\hat{q}$, as in Section 4.2.5. Figure 17 represent the pixel time series $x_{t}\left(i,j\right)$, the pmf $f_{X}\left(x\right)$, and the cumulative Shannon and Tsallis pixel-entropy, while each row represents theoretical pixels.

**Figure 18:**
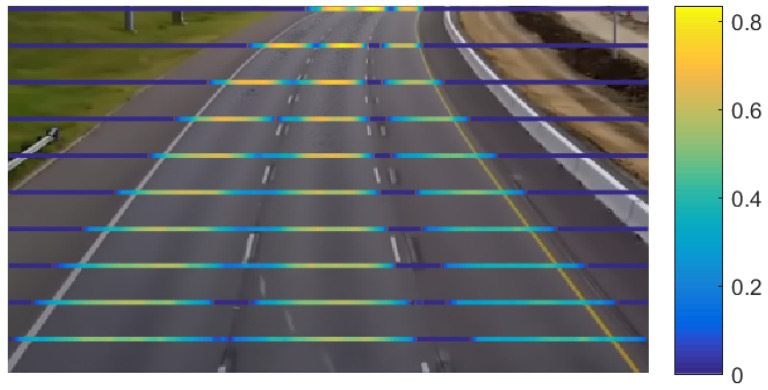
Heat map visualizations of the pixel-entropy at several rows of pixels.

We performed several experiments on different scenarios and pixel positions. A heat map visualization (see Figure 18) allows comparing multiple pixel-entropy levels validating our assumptions about the pixel-entropy behaviors. We observe that, for a traffic load of one vehicle/s per-lane, the pixel-entropy converges around 800 frames (32 s).

### 5.3. Entropic Index Estimation

**Figure 19:**
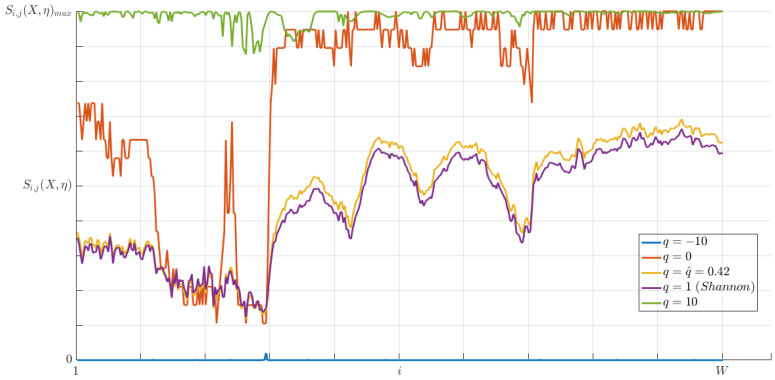
Plots of a particular pixel-entropy vector.

To estimate the entropic index, a finite set of *q*-values ∈R must be given and its evaluation is a very consuming task. Following the methodology in Section 4.2.5, we performed several experiments on different pixel-entropy vectors with different sets of *q*-values and number of microstates (see Figure 19). The experimental results allowed us to determine a finite set of *q*-values based on the Tsallis entropy behavior and the estimated entropic index $\hat{q}=0.42$.

### 5.4. Lane Detection Results

The performance of our algorithm was evaluated comparing the error of the estimated lanes using both Shannon and Tsallis entropy with an algorithm based on trajectories [13,14].

For a detected lane, a good estimate of its center and division lines must reach a high precision, and cover the greatest possible length of the referenced lane. The Absolute Error at Pixel-level (AEP) metric [14] based on the Hausdorff distance was used to compare the lane position error between the positions of a reference lane center/division line and the corresponding estimated positions (see Equation (27)). It is understood that a good lane center or lane division line estimate has a low AEP.

(27) $$AEP\left(L\left(n\right),\hat{L}\left(n\right)\right)=\sum_{a\in L_{k}}\min_{,b\in {\hat{L}}_{k}}∥\left(x_{a}-{\hat{x}}_{b},\;y_{a}-{\hat{y}}_{b}\right)∥$$

where L(n) and $\hat{L}\left(n\right)$ contain the pixel positions $\left(x_{a},y_{a}\right)$ of the $k^{th}$ real lane position and the corresponding estimated positions $\left({\hat{x}}_{a},{\hat{y}}_{b}\right)$, respectively.

Table 2 reports the evaluation of the lane detection stage using the classical metrics (Equations (28)–(30)): Detection Rate (DR), Precision (PRE) and F-Measure (FM). True Positives (TP) is the number of lanes that were detected correctly, False Negative (FN) is the number of lanes that were not detected, and False Positives (FP) is the number of trajectories which are not lanes but detected as lanes by our algorithm. Better results are highlighted in bold.

(28) $$DR=\frac{TP}{TP+FN}$$

(29) $$PRE=\frac{TP}{TP+FP}$$

(30) $$FM=2\times \frac{DR\times PRE}{DR+PRE}$$

Our algorithm detected 38 of 44 lane centers successfully, while trajectory-based achieved only 22 detections. The average detection rate of 86.36% were achieved by the proposed algorithm compared to the 50% of the trajectory-based algorithm. Our algorithm outperforms significantly the detection failures of trajectory-based methods caused by lower number of well-formed trajectories. The average computing time to process a frame takes about 400 μs, and for a pixel row of 420 pixels 40 μs. The total computing time to process 10 pixel rows takes about 65 ms. Our algorithm is 98.62% faster than the one in [14] for processing each frame, and 50.38% faster than the one in [14] for performing an iteration to estimate both the lane centers detection and the lane division lines formation.

**Figure 20:**
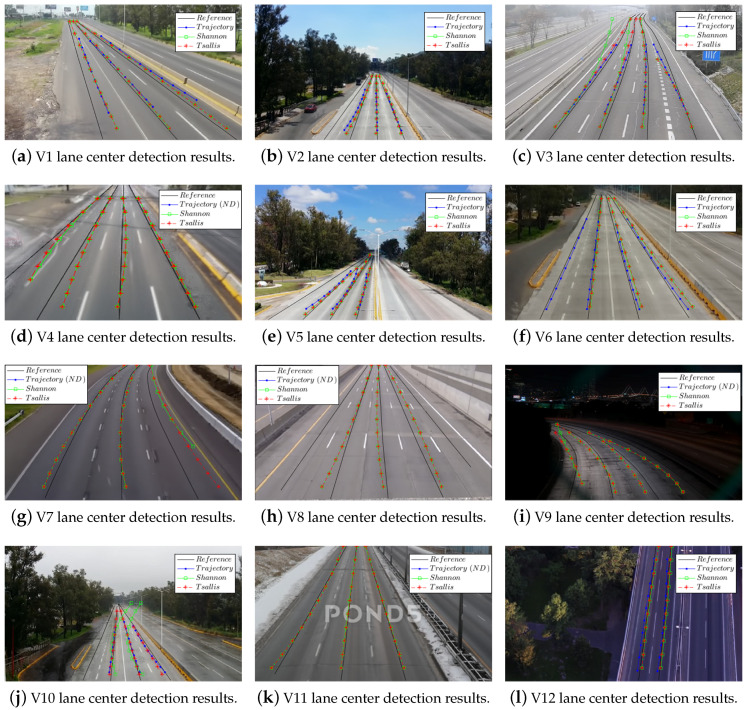
A comparison of the results of trajectory-based with the proposed algorithm for the lane center detection.

Figure 20 shows the qualitative results for the lane detection stage of the scenarios and the second order approximations of the estimated lane centers of our algorithm using Shannon (green) and Tsallis entropy (red), as well as the trajectory-based lane centers (blue). In Figure 20a, lane centers positions of the test methods have almost the same bias; however, the lane length covered by our algorithm is much greater than those covered by trajectory-based algorithms. In Figure 20, the trajectory-based algorithm could not detect any lane center due to the lack of trajectories, mostly because of the camera height and the illumination conditions. In Figure 20, our algorithm could not detect the left lane center due to the lack of traffic flow on this lane. Figure 20 shows a high and slow traffic load per-lane, trajectory-based is not suitable in this situation but our algorithm provides a good estimate of the lane center. Figure 20 shows a notable bias for all tested algorithms in the fourth lane due to the driving behavior on this lane. Finally, for all test scenarios, it is shown that the lane coverage by our algorithm is greater than the trajectory-based algorithm.

Table 3 reports quantitative results of the lane center detection based on the AEP metric (Equation (27)), where the best estimate of each lane center is highlighted in bold. For all tested videos except for the video V6, our algorithm outperforms the trajectory approach with lower AEP of up to 32.33%, less than trajectory AEP with an average of 18.57%. For cases where the performance of our algorithm could not overcome the trajectory-based methods, AEP of up to 11.29% was achieved with an average of 7.34%. For all videos, the lane coverage of our algorithm is greater than the trajectory approach.

Table 4 reports quantitative results of the lane division line formation based on the AEP metric (Equation (27)). Shannon results are omitted because they are similar to Tsallis. A fair comparative against lane marking-based algorithms cannot be achieved because to road marking performs a detection of the static lane division lines, whereas our algorithm performs the dynamic lane division lines formation, and these two types of lane division lines are not necessarily equal in position and in number.

## 6. Discussion

**Test environment**. Several test scenarios with more than 20,000 frames were analyzed, at seven places in different countries, and average traffic loads from 0.40 to 1.69 were used.

**Pixel-entropy**. Shannon and Tsallis entropies were used. Tsallis achieves better results with $q=\hat{q}$. Under a traffic flow of one vehicle/s, entropy values converges in 32 s to stable values.

**Tsallis entropic index *q***. Experimental results show that the range of the *q* for the entropic index estimation is (0+ϵ,1), with ϵ=0.1. For the test videos, *q*-values close to 0.42 were estimated.

**Lane detection**. For the detection stage, our algorithm achieved a high lane detection rate and the highest precision of up to 100% for most scenarios. It was observed that paths with at least five peak vectors were highly reliable.

**Peak matching based on k-means**. *k*-means algorithm was employed to cluster a subset of the peak-vectors (i,j,pk) into *L* lane centers and L+1 lane division lines. *K*-means did not perform well, mostly due to the lack of large amount of samples, noise and outliers. The algorithm cannot perform an automatic detection of lanes number because it requires an input parameter *K* that determines the number of lanes to be found, therefore a priori knowledge of the scenario is necessary.

**Peak matching based on DTW**. the DTW algorithm was employed to select relevant on-road peak vectors for lane centers and lane division lines. It was the algorithm with the best performance achieving the lowest AEP (see Table 3).

**Driving behavior**. It was observed in several videos that on lateral lanes the estimated lane centers are displaced relative to the geometric lane centers due to driver behaviors.

**Limitation**. For very low traffic load in short time periods, the algorithm showed the lowest performance.

## 7. Conclusions

In this paper, a novel and high-performance algorithm for the number of lanes and their centers detection, as well as lane division lines formation based on pixel entropy is presented. To the authors best knowledge, the use of entropy for this purpose has not been done before.

One of the most remarkable features of this algorithm is the automatic detection of all *dynamic lanes* using the DTW algorithm, without knowledge a priori of the number of lanes, making it highly robust to challenging scenarios where the lane and the number of lanes can change.

Experimental results with real data prove that our algorithm outperforms those based on trajectories with respect to computational time for lane description extraction and precision of lane centers significantly, including scenarios with high congestion, partial occlusion, waving vegetation and several perspective views.

Unlike traditional lane division line detection algorithms, which are based on road marking detection, our algorithm performs lane division line formation.

For lane center detection and under the same traffic load conditions, our algorithm shows the lowest computational time.

### Open Issues

1. At pixel domain, it is necessary to reduce the computational time to perform a parallelization of the algorithm.
2. Windowed pixel-entropy can be computed to reduce the FP as result of low traffic load per lane.
3. Other color spaces, such as CIELuv, could be used to study new pixel-entropy behaviors.

## Figures and Tables

**Table 1:** Videos analyzed in this work.

Label	Source	Veh/Sec Avg	Owner/Recording Place	Direction	Weather
V1 [29]	Cell phone	1	Cinvestav/Ringroad, Guadalajara, Mexico	Front	Sunny
V2 [29]	Cell phone	1.45	Cinvestav/Ringroad, Guadalajara, Mexico	Front/Rear	Sunny
V3 [30]	Surveillance	0.63	GRAM-RTM) dataset/M-30, Madrid, Spain	Front/Rear	Foggy
V4 [29]	Cell phone	2	Cinvestav/Ringroad, Guadalajara, Mexico	Front	Cloudy
V5 [29]	Cell phone	1.05	Cinvestav/Ringroad, Guadalajara, Mexico	Front/Rear	Sunny
V6 [29]	Cell phone	1.24	Cinvestav/Ringroad, Guadalajara, Mexico	Front/Rear	Cloudy
V7 [31]	Surveillance	1.6	Alibi/-	Front	Sunny
V8 [32]	Surveillance	1.59	-/-	Front	Sunny
V9 [33]	Surveillance	1.61	-/San Francisco, Unities States	Front	Night
V10 [29]	Cell phone	0.5375	Cinvestav/Ringroad, Guadalajara, Mexico	Front/Rear	Rainy
V11 [34]	Surveillance	1.69	orbitrob/Toronto, Ontario, Canada	Front/Rear	Snowy
V12 [35]	Drone	0.40	sgprolab/Bratislava, Slovakia	Front/Rear	Sunset

**Table 2:** Results of the lane detection stage for Trajectory-based method and our algorithm.

Video	Method	Number of Lanes	TP	FP	FN	Recall	Precision	F-Measure
V1	Trajectory [13,14]	3	3	0	0	100.00	100.00	100.00
	Proposed (Tsallis Entropy)	3	3	0	0	**100.00**	**100.00**	**100.00**
V2	Trajectory [13,14]	8	4	4	0	50.00	100.00	66.66
	Proposed (Tsallis Entropy)	8	5	3	0	**62.50**	**100.00**	**76.92**
V3	Trajectory [13,14]	4	4	0	0	100.00	**100.00**	**100.00**
	Proposed (Tsallis Entropy)	4	4	0	1	**100.00**	80.00	88.88
V4	Trajectory [13,14]	4	0	4	0	0.00	0.00	0.00
	Proposed (Tsallis Entropy)	4	4	0	0	**100.00**	**100.00**	**100.00**
V5	Trajectory [13,14]	8	6	2	0	75.00	100.00	86.85
	Proposed (Tsallis Entropy)	8	6	2	0	**75.00**	**100.00**	**86.85**
V6	Trajectory [13,14]	4	4	0	0	**100.00**	**100.00**	**100.00**
	Proposed (Tsallis Entropy)	4	3	1	1	75.00	75.00	75.00
V7	Trajectory [13,14]	3	0	3	0	0.00	0.00	0.00
	Proposed (Tsallis Entropy)	3	3	0	0	**100.00**	**100.00**	**100.00**
V8	Trajectory [13,14]	5	0	5	0	0.00	0.00	0.00
	Proposed (Tsallis Entropy)	5	3	2	0	**60.00**	**100.00**	**75.00**
V9	Trajectory [13,14]	4	0	4	0	0.00	0.00	0.00
	Proposed (Tsallis Entropy)	3	4	0	0	**100.00**	**100.00**	**100.00**
V10	Trajectory [13,14]	8	3	5	0	37.50	100.00	54.54
	Proposed (Tsallis Entropy)	8	5	3	0	**62.50**	**100.00**	**76.92**
V11	Trajectory [13,14]	3	0	3	0	0.00	0.00	0.00
	Proposed (Tsallis Entropy)	3	3	0	1	**100.00**	**75.00**	**85.71**
V12	Trajectory [13,14]	4	3	1	0	75.00	100.00	86.85
	Proposed (Tsallis Entropy)	4	3	1	0	**75.00**	**100.00**	**86.85**

**Table 3:** AEP results of the lane center detection.

Video	Method	AEP				
		LC *1*	LC *2*	LC *3*	LC *4*	LC *5*
V1	Trajectory [13,14]	1061.49	894.28	1095.70	-	-
	Proposed (Tsallis Entropy)	**967.85**	**682.90**	**769.26**	-	-
V2	Trajectory [13,14]	ND	419.08	265.17	323.35	-
	Proposed (Tsallis Entropy)	ND	**284.02**	**230.76**	**258.87**	-
V3	Trajectory [13,14]	1018.55	970.79	977.18	**974.85**	-
	Proposed (Tsallis Entropy)	**804.00**	**795.89**	**802.65**	1098.95	-
V4	Trajectory [13,14]	ND	ND	ND	ND	-
	Proposed (Tsallis Entropy)	**678.79**	**966.92**	**735.91**	**753.34**	-
V5	Trajectory [13,14]	ND	475.15	384.46	327.25	-
	Proposed (Tsallis Entropy)	ND	**339.15**	**260.15**	**277.92**	-
V6	Trajectory [13,14]	**714.82**	672.24	**777.29**	**780.85**	-
	Proposed (Tsallis Entropy)	ND	**626.93**	794.00	825.17	-
V7	Trajectory [13,14]	ND	ND	ND	-	-
	Proposed (Tsallis Entropy)	**829.00**	**705.52**	**1314.58**	-	-
V8	Trajectory [13,14]	ND	ND	ND	ND	ND
	Proposed (Tsallis Entropy)	ND	**840.84**	**843.65**	**880.25**	ND
V9	Trajectory [13,14]	ND	ND	ND	ND	-
	Proposed (Tsallis Entropy)	**516.68**	**404.85**	**616.29**	**634.07**	-
V10	Trajectory [13,14]	ND	385.04	356.05	**443.58**	-
	Proposed (Tsallis Entropy)	ND	**289.17**	**294.37**	496.21	-
V11	Trajectory [13,14]	ND	ND	ND	-	-
	Proposed (Tsallis Entropy)	**710.44**	**643.12**	**662.99**	-	-
V12	Trajectory [13,14]	699.69	673.42	-	-	-
	Proposed (Tsallis Entropy)	**669.46**	**658.99**	-	-	-

ND: Not Detected.

**Table 4:** AEP results of the lane division lines formation.

Video	Method	AEP					
		LD *1*	LD *2*	LD *3*	LD *4*	LD *5*	LD *6*
V1	Proposed (Tsallis Entropy)	1041.62	675.14	752.10	819.16	-	-
V2	Proposed (Tsallis Entropy)	438.49	ND	258.76	275.28	426.68	-
V3	Proposed (Tsallis Entropy)	1086.02	809.18	790.08	1179.00	927.75	-
V4	Proposed (Tsallis Entropy)	537.44	907.93	831.06	910.03	1110.99	-
V5	Proposed (Tsallis Entropy)	491.55	ND	327.51	289.59	343.03	-
V6	Proposed (Tsallis Entropy)	875.31	ND	770.89	725.34	952.23	-
V7	Proposed (Tsallis Entropy)	1335.98	759.15	841.04	1240.45	-	-
V8	Proposed (Tsallis Entropy)	ND	834.81	646.83	739.26	ND	866.37
V9	Proposed (Tsallis Entropy)	920.93	431.25	471.96	556.89	523.27	-
V10	Proposed (Tsallis Entropy)	454.86	ND	281.06	442.99	603.41	-
V11	Proposed (Tsallis Entropy)	1168.24	702.76	626.33	1379.81	-	-
V12	Proposed (Tsallis Entropy)	1167.55	603.92	828.40	-	-	-
